# Amyloid Goiter Secondary to Ulcerative Colitis

**DOI:** 10.1155/2016/3240585

**Published:** 2016-03-08

**Authors:** Bunyamin Aydin, Yavuz Savas Koca, Tugba Koca, Ihsan Yildiz, Sevda Gerek Celikden, Metin Ciris

**Affiliations:** ^1^Division of Endocrinology and Metabolism, Department of Internal Medicine, Suleyman Demirel University, School of Medicine, 32200 Isparta, Turkey; ^2^Department of General Surgery, Suleyman Demirel University, School of Medicine, 32200 Isparta, Turkey; ^3^Department of Pediatric Gastroenterology, Hepatology and Nutrition, Suleyman Demirel University, School of Medicine, 32200 Isparta, Turkey; ^4^Department of Pathology, Suleyman Demirel University, School of Medicine, 32200 Isparta, Turkey

## Abstract

Diffuse amyloid goiter (AG) is an entity characterized by the deposition of amyloid in the thyroid gland. AG may be associated with either primary or secondary amyloidosis. Secondary amyloidosis is rarely caused by inflammatory bowel diseases. Secondary amyloidosis is relatively more common in the patients with Crohn's disease, whereas it is highly rare in patients with ulcerative colitis. Diffuse amyloid goiter caused by ulcerative colitis is also a rare condition. In the presence of amyloid in the thyroid gland, medullary thyroid cancer should be kept in mind in the differential diagnosis. Imaging techniques and biochemical tests are not very helpful in the diagnosis of secondary amyloid goiter and the definitive diagnosis is established based on the histopathologic analysis and histochemical staining techniques. In this report, we present a 35-year-old male patient with diffuse amyloid goiter caused by secondary amyloidosis associated with ulcerative colitis.

## 1. Introduction

Amyloidosis is a general term describing a group of diseases characterized by the deposition of an insoluble, proteinaceous, amorphous, and eosinophilic material, called amyloid, in the extracellular matrix of some organs and tissues [1]. Many proteins enter the so-called amyloid state, in which they form elongated fibers, with spines consisting of many-stranded *β* sheets. The operational definition of amyloid, which has been adopted by the community of pathologists, is that the fibers are unbranched, usually extracellular, and found in vivo; in addition, the fibers bind the dye Congo Red and then show green birefringence when viewed between crossed polarizers [2]. By this definition, fewer than 25 amyloid-forming proteins have been identified and associated with serious diseases, including amyloid-*β* peptide (A*β*) with Alzheimer's disease (AD), islet amyloid polypeptide (IAPP) with diabetes type 2, and prion protein (PrP) with the spongiform encephalopathies [3].

Primary amyloidosis is characterized by the deposition of amyloid L (AL), which is the major fibrillary protein originating from the light chain components of immunoglobulins, whereas secondary amyloidosis is characterized by the deposition of amyloid A (AA), which constitutes a portion of the acute-phase serum amyloid A (SAA) protein produced by the liver during inflammation. Amyloid goiter is clinically defined as the deposition of amyloid in the thyroid gland in such quantities as to produce an enlargement in the gland. To date, an approximate total of 85 cases of amyloid goiter have been reported in the literature. Deposition of amyloid in the thyroid gland may coexist with systemic amyloidosis, with medullary thyroid carcinoma, and less commonly with primary amyloidosis involving the thyroid gland. Systemic amyloidosis may be accompanied by the involvement of the thyroid gland alone [4]. The progressive increase in the thyroid volume in amyloidosis mostly does not impair the thyroid function [5].

Our patient presented with diffuse intrathyroidal thyroid amyloidosis that developed on the basis of secondary amyloidosis caused by ulcerative colitis.

## 2. Case Report

**Figure 1:**
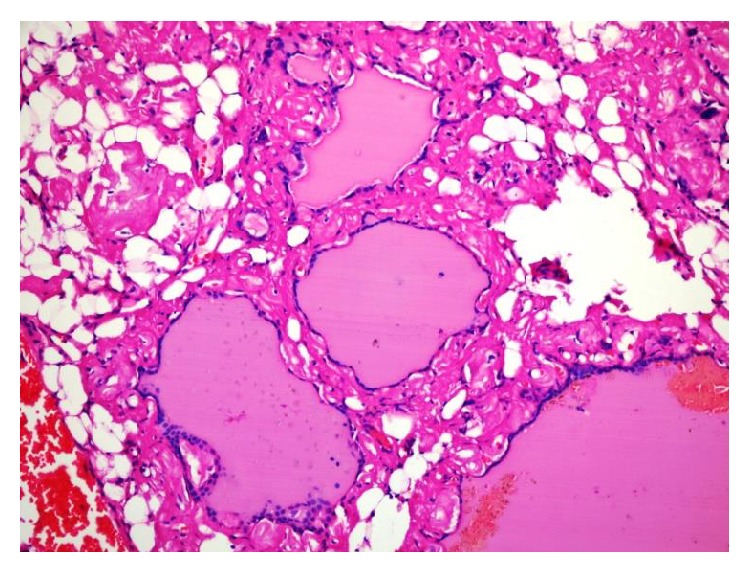
Diffuse eosinophilic amorphous material interfollicular sites (hematoxylin and eosin, 400x).

**Figure 2:**
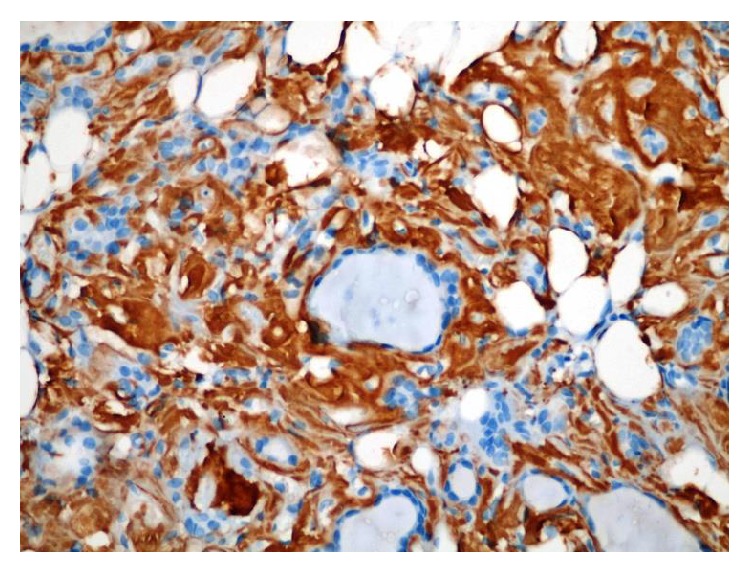
Immunohistochemical method to stain amyloid (400x).

**Figure 3:**
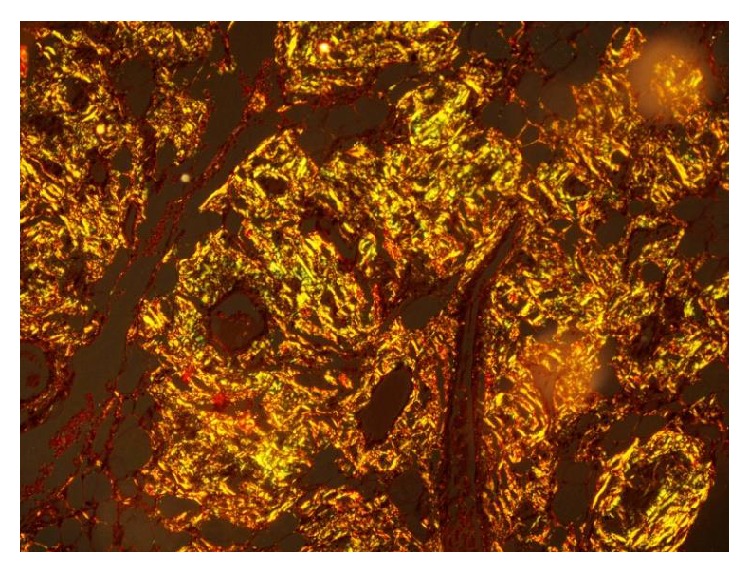
Section of thyroid gland with positive apple-green birefringence to Congo Red stain under polarized light (400x).

The 35-year-old male patient presented to our clinic with the complaints of headache, sore throat, a one-month history of a rapidly growing mass in the neck, and weight loss. At presentation, the patient had been followed up due to the diagnosis of ulcerative colitis for the last 10 years. Physical examination revealed a pain in the neck and a 1 cm nodule in the thyroid isthmus on palpation. Moreover, the breath sounds were decreased in the right lung. Laboratory tests were as follows: white blood cell: 3800/mm^3^, hemoglobin: 12.6 g/dL, platelet: 159,000/L, C-reactive protein (CRP): 25 mg/L (normal, 0 to 1 mg/dL), glucose: 90, urea: 47, creatinine: 1.07 mg/dL (0.84–1.25), aspartate transaminase (AST): 26 U/L (8–40), alanine transaminase (ALT): 43 U/L (5–35), sodium: 139 mmol/L (136–146), potassium: 4.7 mmol/L (2.5–4.5), triglyceride: 342 mg/dL (35–150), HDL: 35 mg/dL (40–60), total cholesterol: 302 mg/dL (120–200), and sedimentation: 76/h. The cardiothoracic ratio was increased on the PA-AC graph. Pericardial effusion was detected during the consultations with the relevant departments. During the investigation of the etiology of the pericardial effusion, nephrotic-range proteinuria (3.6 gr/day) was detected. Subcutaneous biopsy was performed on suspicion of amyloidosis in the etiology of proteinuria but no sign of amyloidosis was found in the immunohistochemical analysis. Duodenal and rectal mucosal biopsies were performed but amyloid was detected only in the rectal mucosa. The presence of amyloid was confirmed by the appearance of Congo Red and apple-green birefringence and immunohistochemical staining patterns. Thyroid function tests were as follows: Ft3: 2.14 pg/mL (normal, 2.5 to 3.9 pg/mL), Ft4: 1.01 ng/dL (0.61–1.12), TSH: 0.29 *μ*IU/mL (0.34–4.2), anti-thyroid peroxidase (Anti-TPO): 28 IU/mL (0–35), and antithyroglobulin (AntiTG): 110 IU/mL (0–115). On ultrasonography, both thyroid parenchymata were heterogeneous, whereas a 12 × 8 mm heterogeneous, degenerated, mixed nodule was detected in the isthmus and a 13 × 8 mm cystic nodule was detected in the inferior segment of the right lobe. Thyroid scintigraphy revealed low thyroid activity and increased background activity in the thyroid area. Fine needle aspiration of the thyroid gland revealed a 12 × 8 mm mixed nodule with benign cytological results in the isthmus and an 18 mm cystic nodule at the junction of the left lobe with the isthmus. Thyroid scintigraphy was normal. In the follow-up period, total thyroidectomy was performed due to euthyroidism. Pathological analysis revealed amyloid deposition in the entire thyroid gland and deposition of amorphous eosinophilic material between the follicular structures. Amyloid was histochemically stained with PAS and Congo Red stains and was immunohistochemically classified as AA (Figures 1, 2, and 3). The patient has been followed up for the last 15 months, and he is currently undergoing the treatment of levothyroxine intoxication and is in remission for amyloidosis that developed secondary to ulcerative colitis.

## 3. Discussion

Amyloid goiter (AG) is a rare thyroid lesion although it was described a long time ago [5]. AG may be associated with either primary or secondary amyloidosis. The amyloid goiter associated with secondary amyloidosis that occurs secondary to inflammatory diseases including tuberculosis, bronchiectasis, cystic fibrosis, ulcerative colitis, Crohn's disease, rheumatoid arthritis, and ankylosing spondylitis is more common. Kimura et al. reported that 90% of their AG patients presented with secondary amyloid and AG occurred secondary to rheumatoid arthritis in most of these cases [6]. Amyloidosis arising from inflammatory bowel diseases is a very rare but serious complication. In a cohort study by Greenstein et al., secondary amyloid deposition was detected in 15 of 1,709 patients diagnosed with Crohn's disease and in 1 of 1,341 patients with ulcerative colitis. Amyloidosis with renal involvement is more common in inflammatory bowel diseases. However, thyroid involvement is less common [7, 8]. Our patient presented with diffuse intrathyroidal thyroid amyloidosis that developed on the basis of secondary amyloidosis caused by ulcerative colitis, which is a highly rare condition. AG rarely presents as the first manifestation of systemic amyloidosis. Conversely, AG may present as diffuse enlargement of the thyroid gland within several weeks of months. This enlargement may be accompanied by stridor hoarseness, dyspnea, dysphagia, and regional lymphadenopathy. Our patient had a mass in the neck which had been rapidly growing for the last one month. In patients with amyloid goiter, the thyroid function tests often indicate euthyroid. In addition, cases of simultaneous occurrence of hyper- and hypothyroidism have also been reported [9].

Fine needle aspiration biopsy is a simple, sensitive, and safe method used in the diagnosis of thyroid diseases. Nevertheless, in patients with amyloid goiter, the definitive diagnosis relies on the histological analysis of the thyroid gland that is excised. Moreover, histochemical staining techniques including Congo Red, thioflavin T, and crystal violet stains are used for confirming the presence of amyloid. Among these, Congo Red is the most commonly used staining method, which imparts an apple-green birefringence under polarized light and is accepted as a pathognomonic property of amyloid. AA can be differentiated from other amyloid proteins via immunohistochemical analysis [10]. In our patient, the histopathologic analysis revealed intrathyroidal amyloid deposition in the entire thyroid gland. The presence of amyloid was confirmed by the appearance of Congo Red and apple-green birefringence under polarized light.

In conclusion, amyloid goiter should be kept in mind in the patients suspected of primary or secondary amyloidosis, particularly in the patients presenting with a rapidly growing goiter and obstructive symptoms.
